# Maternal and Perinatal Outcomes in Patients with Suspected COVID-19 and Their Relationship with a Negative RT-PCR Result

**DOI:** 10.3390/jcm9113552

**Published:** 2020-11-04

**Authors:** Yolanda Cuñarro-López, Óscar Cano-Valderrama, Pilar Pintado-Recarte, Ignacio Cueto-Hernández, Blanca González-Garzón, Santiago García-Tizón, Julia Bujan, Ángel Asúnsolo, Miguel A. Ortega, Juan Antonio De León-Luis

**Affiliations:** 1 Department of Public and Maternal and Child Health, School of Medicine, Complutense University of Madrid, 28040 Madrid, Spain; yolanda.cunarro@gmail.com (Y.C.-L.); ppintadorec@yahoo.es (P.P.-R.); ignaciocuetohernandez@gmail.com (I.C.-H.); b.glez-garzon@hotmail.com (B.G.-G.); gineteca@gmail.com (S.G.-T.); jaleon@ucm.es (J.A.D.L.-L.); 2 Department of Obstetrics and Gynecology, University Hospital Gregorio Marañón, Madrid 28009, Spain; 3 Health Research Institute Gregorio Marañón, 28009 Madrid, Spain; 4 Department of Surgery, Complutense University of Madrid, 28009 Madrid, Spain; oscarcanovalderrama@hotmail.com; 5 Department of Medicine and Medical Specialties, Faculty of Medicine and Health Sciences, University of Alcalá, Alcalá de Henares, 28801 Madrid, Spain; mjulia.bujan@uah.es; 6 Ramón y Cajal Institute of Healthcare Research (IRYCIS), 28034 Madrid, Spain; angel.asunsolo@uah.es; 7 University Center for the Defense of Madrid (CUD-ACD), 28047 Madrid, Spain; 8 Department of Surgery, Medical and Social Sciences, Faculty of Medicine and Health Sciences, University of Alcalá, Alcalá de Henares, 28801 Madrid, Spain; 9 Department of Epidemiology & Biostatistics, Graduate School of Public Health and Health Policy, University of New York, New York, NY 10027, USA

**Keywords:** SARS-CoV-2, COVID-19, pregnancy, maternal-perinatal outcomes, RT-PCR negative result

## Abstract

This study was designed to examine maternal-perinatal outcomes in pregnant women with suspected coronavirus disease 2019 (COVID-19) according to the result of a real-time reverse transcription polymerase chain reaction (RT-PCR) test and to investigate possible variables that could be useful for predicting a negative RT-PCR result. Participants of this retrospective cohort study were obstetrics patients with suspected COVID-19 who underwent an RT-PCR test in a tertiary hospital in Madrid, Spain. Maternal-perinatal features were analysed according to the results of this test. Clinical, radiological and analytical characteristics that could be associated with a negative result were also explored. In a final subgroup analysis, patients were included if they had pneumonia and a negative test result for the virus. Out of the 111 obstetric patients with suspected COVID-19 that were enrolled, 38.7% returned a negative result. In this RT-PCR-negative group, we recorded lower rates of pneumonia (21.4% vs. 45.6%, *p* = 0.009), severe or critical clinical features (4.7% vs. 11.8% and 0.0% vs. 5.9%, *p* = 0.02, respectively), lower lactate dehydrogenase (LDH) levels (168 UI/L vs. 224.5 UI/L, *p* = 0.003), a greater need for maternal treatment (60.3% vs 24.4%, *p* < 0.001), a reduced need for oxygen therapy (2.4% vs 28.8%, *p* < 0.001) and a lower rate of intensive care unit admission (0.0% vs. 3.7%, *p* = 0.046) than the RT-PCR-positive group. While no differences were found in other variables, the monocyte count was higher (946.2/μL vs. 518.8/μL, *p* = 0.022) in this group. The predictive model for a negative test result included the monocyte count, LDH level and no need for oxygen therapy. This model was able to identify 73.5% of patients with a negative RT-PCR result. Only 11% of the patients with pneumonia testing negative for the virus had IgG antibodies against severe acute respiratory syndrome coronavirus 2 (SARS-CoV-2). The proportion of pregnant women with suspected COVID-19 and a negative RT-PCR result was nearly 39%. In these patients, the symptoms were mild and the systemic severity of the disease was lower. The monocyte count, LDH level and no need for oxygen therapy were the factors that were more related to a negative test result in this group. These variables could be used to guide the management of patients with suspected COVID-19, mainly while waiting for RT-PCR results or in settings where this test is not available.

## 1. Introduction

Severe acute respiratory syndrome coronavirus 2 (SARS-CoV-2), the pathogen that causes coronavirus disease 2019 (COVID-19), was first reported in Wuhan, China, and has spread all around the world. On 20 September 2020, over 30.6 million COVID-19 infections and 950,000 deaths have been reported [1].

A diagnosis of SARS-CoV-2 infection is usually made by detecting the RNA of the virus using real-time reverse transcription polymerase chain reaction (RT-PCR) on a nasal swab, sputum or throat swab. Nevertheless, this technique has significant limitations as it requires certified laboratories, expensive equipment and trained technicians. Moreover, as respiratory secretions may be quite variable in their composition, the adequacy of sampling efforts may also vary [2] and sensitivity and specificity can also be a problem, as false negative results have been reported [3]. Finally, another limitation of this technique is that its results may sometimes take several hours to arrive. This occurred mostly at the beginning of the pandemic and still does in busy faculties. Avoiding RT-PCR result delays is important for pregnant patients who present to an emergency department because they are in labour or because they need an urgent or emergency obstetrical procedure.

Recently, the World Health Organization introduced the use of faecal samples or rectal swabs for the determination of SARS-CoV-2 [2]. Several published works, such as that of Zheng et al. [4] and Santos et al. [5], have suggested that the detection of viral RNA in these samples takes longer than in respiratory tract specimens [2,4]. Therefore, this technique should be used for diagnostic confirmation in those patients with symptoms suggestive of COVID-19 and a negative RT-PCR result obtained in respiratory tract specimens.

Over the past few months, the detection of specific antibodies against SARS-CoV-2 is also being used to diagnose COVID-19. These tests have several advantages over RT-PCR. For example, their results are faster and their high sensitivity (88.7%) and specificity (90.3%) have been reported by some authors [3]. However, other studies have found that only 40% of patients develop antibodies within 1 week of the disease onset [6]. Furthermore, not all these tests are available in the countries where the pandemic has struck, such as low-income countries with a low human development index.

Taking into account the limitations of the available diagnostic tests and that clinical symptoms can be unspecified and mild, it is important to study the analytical, radiological and clinical features that could be suggestive of COVID-19.

This study sought to analyse maternal and perinatal outcomes (MPO) and clinical and analytical findings in patients with suspected COVID-19 during gestation, labour and delivery, and the puerperium period. Relationships between these features and the RT-PCR COVID-19 test result were also examined to develop a model that could predict a negative RT-PCR result. This model could be useful while the result of the RT-PCR is pending or in those environments where RT-PCR is not available.

## 2. Patients and Methods

### 2.1. Patient Cohort and Study Design

An observational, analytic, retrospective cohort study with a longitudinal follow-up was performed. This study was performed according to the Strengthening the Reporting of Observational Studies in Epidemiology (STROBE) guidelines [7].

This study was performed in a tertiary centre located in Madrid, where community transmission of the virus has been described and a high incidence, morbidity and mortality of this disease have been reported. On 24 September 2020, 213,709 cases of SARS-CoV-2 infection and 9213 deaths had been reported [8]. This hospital is a referral centre for obstetrics, maternal-fetal medicine and neonatology, with approximately 5100 deliveries in 2018 and 2019. On 22 March 2020, urgent and emergent obstetric and neonatal attention was modified in the Madrid region due to the COVID-19 pandemic [9]. With the new model, the obstetric activity of 4 hospitals was transferred to this centre, which meant 358 more deliveries per month.

Inclusion criteria were obstetrics patient (pregnant, in labour or puerperium) with suspected COVID-19 that attended our hospital. All of these patients were assessed using high-throughput sequencing or an RT-PCR assay of nasal or pharyngeal swab specimens. Based on the results obtained using RT-PCR, the patients were divided into two groups: an RT-PCR-positive group and an RT-PCR-negative group.

Patients with a non-conclusive RT-PCR result, those patients who did not undergo obstetric follow-up in our hospital and asymptomatic patients with SARS-CoV-2 infections were excluded.

Recruitment was performed between 10 March 2020 (first obstetric patient with COVID-19 in our center) and 12 May 2020 since, as of that date, the incidence of obstetric patients with symptoms suggestive of COVID-19 decreased significantly due to being at the end of the first wave of the pandemic in the community of Madrid. Opportunistic population screening of asymptomatic patients who were admitted for delivery care began at the end of April and they had been excluded from work.

Data collection was performed with a standard form. The variables that were collected for each patient were as follows: maternal features, such as race, maternal age, use of tobacco, maternal morbidities and body mass index (BMI) (kg/m^2^); obstetrics features, including gestational morbidities, parity, onset of symptoms in pregnancy or puerperium and gestational age (GA) at triage; maternal symptoms and signs, such as fever (>37.3 °C), cough, shortness of breath, diarrhoea, temperature, oxygen saturation and breathing frequency at triage; complementary maternal studies, including the results of the oral swab, presence or absence of pneumonia and blood sampling for leukocytes, lymphocytes, monocytes, platelets and lactate dehydrogenase (LDH) at triage; maternal treatments, such as antiviral, antibiotics, antirheumatics or anticoagulants and MPOs, including the need for oxygen therapy (if oxygen saturation of room air at rest was <94%) [10], maternal admission to an intensive care unit (ICU), maternal mortality, GA at delivery, mode of delivery (C-section or vaginal delivery) and neonatal birthweight; Apgar score at five minutes; admission to the neonatal intensive care unit (NICU), neonatal mortality and vertical transmission in the first 24 h post birth.

According to the seventh version of the guidelines on the Diagnosis and Treatment of COVID-19 by the National Health Commission of China [11], COVID-19 severity is classified as follows:

1. Mild cases—the clinical symptoms were mild and there was no sign of pneumonia when imaged.
2. Moderate cases—fever and respiratory symptoms with radiological findings of pneumonia.
3. Severe cases—any of the following conditions:
  a. Respiratory distress (respiratory rate of ≥30 per min).
  b. Oxygen saturation on room air at rest of ≤93%.
  c. Partial pressure of oxygen in arterial blood/fraction of inspired oxygen ≤ 300 mmHg.
4. Critical cases—any of the following conditions:
  a. Respiratory failure and requiring mechanical ventilation.
  b. Shock.
  c. Patients with another organ failure that requires ICU care.

A descriptive study of all the patients included in the study was performed. Furthermore, we performed an analytical study comparing the MPO based on the RT-PCR result and a predictive multivariate analysis to identify the clinical, radiological or analytical variables that were related to a negative RT-PCR result. Finally, a subgroup analysis was performed in those patients with radiological signs of pneumonia and a negative result from the RT-PCR test. These patients were submitted to a new study involving RT-PCR and tests for IgG antibodies against SARS-CoV-2 four weeks after the clinical symptoms had disappeared.

The detection of serum IgG antibodies against the SARS-CoV-2 nucleocapsid protein was carried out in the ARCHITECT analyser using Abbott’s SARS-CoV-2 IgG assay (Abbott, Abbott Park, IL, USA) following the manufacturer´s instructions. The assay is based on a chemiluminescent microparticle immunoassay and determinations were considered negative or positive depending on whether the results were <1.4 or ≥1.4, respectively (cut-off index value).

### 2.2. Data Analysis

Data obtained from the study were included in a Microsoft Office Excel database, version 16.42 (Microsoft, Redmond, WA, USA) and the statistical analysis was performed with Stata 13.1 (StataCorp LLC, College Station, TX, USA). Differences with *p* < 0.05 were considered statistically significant. Quantitative variables were expressed as the mean (interquartile range or 95% confidence interval (CI)) and categorical variables as the number of patients and rates (%) (CI 95%). Univariate analysis was performed using Fisher’s exact test, chi-squared test or Student’s *t*-test, as appropriate. Variables that were related to a negative RT-PCR result (clinically or statistically significant differences between groups) were included in the multivariate analysis that was performed with logistic regression. The final regression model was chosen according to the Akaike information criterion, the area under the curve and the Hosmer–Lemeshov *p*-value after analysing all the possible models.

### 2.3. Ethical Approval

The authors declare that they have no conflict of interest. All procedures performed in studies involving human participants were in accordance with the ethical standards of the institutional and/or national research committee and with the 1964 Helsinki declaration [8] and its later amendments or comparable ethical standards. Consent forms were obtained and the research was approved by the Institutional Review Board (Code: COVID-GESTA).

## 3. Results

**Figure 1:**
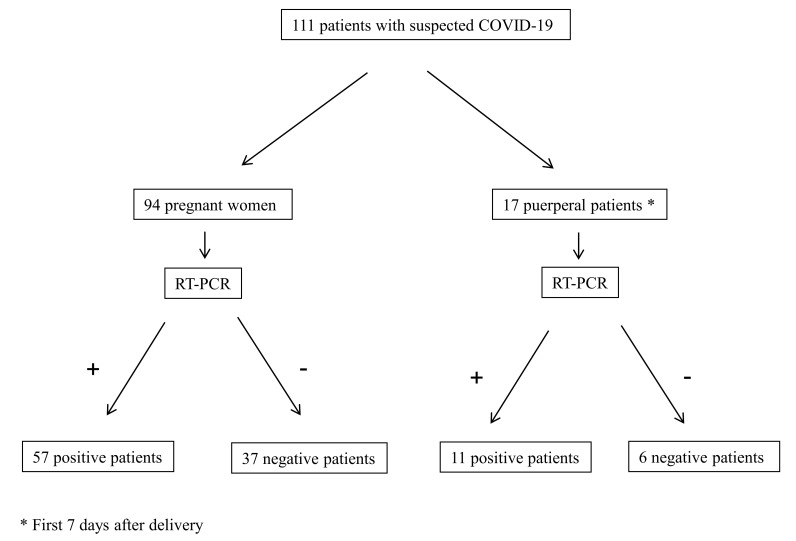
Flowchart of the patients included. COVID-19: coronavirus disease 2019, RT-PCR: reverse transcription polymerase chain reaction.

A total of 111 patients with suspected COVID-19 that underwent RT-PCR testing during pregnancy, labour or puerperium were included in the study. During the study period, 1026 labours were attended in our hospital; this means that COVID-19 was suspected in one out of nine labours. Out of 111 patients, 94 (84.7%) were pregnant women and 17 (15.3%) developed the clinical symptoms during the first days after delivery, while 68 (61.3%) of the patients had a positive RT-PCR result and 43 (38.7%) had a negative one. The flowchart of the patients included can be seen in Figure 1.

At the end of the study period, 52 out of the 111 patients (46.9%) had already given birth, where 39 (75.0%) of these were in the group of patients with a positive RT-PCR result and 13 (25.0%) were in the negative RT-PCR result group. Table 1 shows the MPO of the patients included in the study in terms of the overall same and for each study group. Clinically, 95 (85.6%) patients had at least one symptom at diagnosis, with fever and cough being the most common ones. C-section was the birth route for half the cases, principally for maternal clinical worsening (40.9%), and non-vertical transmission was observed during the first 24 h.

The RT-PCR negative group showed a statistically significant difference in terms of higher monocyte counts and lower LDH levels, radiological pneumonia, maternal admission in the ICU, maternal treatment and a need for oxygen therapy. Non-statistically significant differences were found for the rest of the collected variables.

Table 2 shows the distribution of the women according to the severity of the COVID-19 for the overall sample and between the study groups. For the overall sample, 71 (64.0%) had a mild form of the disease and were statistically more frequent in the RT-PCR-negative group. The multivariate analysis included the monocyte count, LDH level, need for oxygen therapy, pneumonia and maternal admission to the ICU. These last two variables were excluded in the final predictive model, which was able to properly classify 73.5% of the patients (75% sensitivity; 72.5% specificity; predictive negative value of 80.6% and an area under the curve of 0.79). The final regression equation was

(PCRnegative = 1/X) = 1/1 + e − (1.37 + 0.001 * monocytes-0.012 * LDH-1.815 * oxygen)(1)

which had an *R*^2^ of 0.19. With this model, the probability of a negative RT-PCR result can be estimated. For example, a pregnant woman with a monocyte count of 500/μL, an LDH level of 300 UI/L and needing oxygen therapy would have a probability of 3.2% (CI 95%: 0.3–26.0%) of an RT-PCR negative result. On the other hand, a patient with a monocyte count of 1200/μL, an LDH level of 100 UI/L and without oxygen therapy would have a probability of 83.6% (CI 95%: 47.8–96.6%) of an RT-PCR negative result.

Finally, there were nine patients with symptoms of COVID-19, radiological findings of pneumonia and had a negative RT-PCR result. From this group, eight (88.9%) had negative results for both antibodies and the RT-PCR and one (11.1%) had a negative RT-PCR result and was positive for antibodies four weeks after the symptoms’ disappearance.

## 4. Discussion

According to our results, obstetric patients with suspected COVID-19 represented 9.2% of the labours in the study period, where nearly 40% of them had a negative RT-PCR result. For this group of patients, a significantly lower proportion of pneumonia and severe or critical COVID-19, lower LDH levels, less need for maternal treatment or oxygen therapy and fewer patients requiring admission to the ICU were found. On the other hand, the monocyte count was higher. Overall, fever and cough were the most common symptoms, but there were no statistically significant differences between the RT-PCR groups. Furthermore, the overall rate of C-sections and prematurity was higher compared to clinical practice but similar in both groups.

Pregnant COVID-19 positive women accounted for nearly 10% of the labours, where similar results were found by Sutton et al. [12,13] in New York City, who found that 13.5% of patients admitted for delivery in a universal screening tested positive for SARS-CoV-2; both cities (Madrid and New York City) had a high incidence of COVID-19 during the pandemic period.

Nearly one in three patients had a negative RT-PCR result and 21.4% of these patients showed pneumonia in the chest X-ray. Li et al. [14] have also studied this issue, finding that 42.6% of non-obstetric adult patients with pneumonia had a negative RT-PCR result. Based on the specific IgG antibody results (only 11% positive), this suggests that, even during the pandemic period, other pathogens could be responsible for pneumonia. Another possible explanation would be the low sensitivity and specificity of the diagnostic tests.

When analysing the country of origin of the patients, nearly half of the women were from Latin America (Table 1), which is three times higher than what Blagoeva et al. report, with 17.5% of foreign patients in Spain for 2015 at the time of delivery [15]. Although there were no differences between the RT-PCR groups, it is important to consider that foreign pregnant women had a higher risk of maternal morbidity and mortality, which was dependent on the country of origin [16,17].

It is also relevant to consider that the maternal BMI of our COVID-19 patients was high and the relationship between obesity and COVID-19 has previously been described, where obesity, hypertension and diabetes were associated with a high risk of severe COVID-19 that needed hospital admission and mechanical ventilation [18,19].

Although no maternal deaths were observed in the study, the maternal-perinatal binomial had a high incidence of morbid events, as previously described [20]. For example, the C-section rate was 44.2% because of the important maternal worsening, prematurity was observed in 28.9% of the neonates, 32.7% of the neonates were admitted to the NICU and the neonatal mortality was 4.2%. This morbidity could be related to the quick respiratory worsening of the mother, which forced an urgent and early finishing of the pregnancy.

When patients with a positive or negative RT-PCR result were compared, significant differences were found in the radiological and laboratory tests, such as the platelet count, monocyte count and LDH level. Several causes could explain these differences; for example, the high affinity of SARS-CoV-2 for the lower respiratory tract would manifest as ground-glass opacities and consolidations with a peripheral and posterior lung distribution [21]. However, COVID-19 is not only a respiratory syndrome. Systemic infection with a significant impact on the haematopoietic and haemostasis systems has also been described. This systemic infection induces an excessive inflammatory response that is associated with high levels of circulating cytokines, severe lymphopenia and substantial mononuclear cell infiltration in the lungs, heart, spleen, lymph nodes and kidneys [22]. This inflammatory response is the reason for the severe alterations that can be seen on the laboratory tests of the patients with COVID-19 (decrease in the number of lymphocytes, haemoglobin, platelets and monocytes and elevated LDH and aminotransferase levels) [23].

Depending on the RT-PCR result (negative vs. positive), clinically significant differences were found for the C-section rate (30.8% vs. 48.7%), prematurity (15.4% vs. 33.3%), NICU admission (23.1% vs. 35.9%) and neonatal mortality (0.0% vs. 7%), respectively. These differences were not statistically significant, which was probably due to the sample size.

Table 2 shows that most patients had mild (64%) or moderate (23.4%) symptoms, with only 14% of the women with severe or critical COVID-19. Severity in our patients was higher than previously reported from non-pregnant populations [24,25] or asymptomatic pregnant women finishing their pregnancies [9]. This increased severity could be explained by the inclusion of patients with suspected COVID-19, instead of performing a universal screening. Although the mortality has been reported to be around 50% in the non-pregnant population with critical COVID-19 [23,24], no maternal mortality was observed in our series. The lower maternal age of the pregnant patients could explain this low mortality. However, these results should be taken with caution because other studies [26] reported 7 cases of maternal mortality among 9 pregnant women with severe COVID-19 disease in Iran and Di Mascio et al. [27] published 3 cases of maternal mortality between 388 pregnant women from high-income and middle-income countries.

The proportion of patients with mild cases was higher in the group of patients with a negative RT-PCR result (79.1% vs. 52.4%). It would be interesting to study the IgG antibodies for SARS-CoV-2 in all the patients with a negative RT-PCR result. Nevertheless, this test is not widely available and we could only perform it on those patients with pneumonia and a negative RT-PCR result. Only one of them (11%) had SARS-CoV-2 antibodies; therefore, it would be possible that these cases of pneumonia had been caused by other pathogens. Patients with severe or critical symptoms usually displayed a positive result from the RT-PCR test. This finding was previously observed [28]. They reported that patients with a higher viral load in the lower respiratory tract displayed a more severe version of the disease.

In the predictive model, we observed that monocyte count, LDH level, and oxygen therapy could be useful factors to take into account to predict a negative value of the RT-PCR. This model properly classified 73.5% of the patients. The variables included in the model give us information about the hematologic, hepatic and respiratory situation of the systemic affectation of the patient. Interestingly, all three variables are cheap, quick and easy to obtain without performing an invasive technique that could put the health workers at risk. Therefore, these variables could be obtained, even in low-resource centres of countries with low socioeconomic development.

Nowadays, the COVID-19 status in Spain is usually studied using RT-PCR and IgG antibodies. However, many centres around the world do not have access to these techniques due to their cost and the delay of their results. Hence, the predictive model will be useful in these centres, as they will be able to predict the RT-PCR result with these easy-to-obtain variables. 

The main strength of this study was the large number of obstetric patients with suspected COVID-19 that were nursed in a single centre in Madrid during a short period. Furthermore, most studies previously published included only patients with COVID-19 that were confirmed using RT-PCR. Meanwhile, our study analysed and compared the differences and similarities between those patients with a positive and a negative RT-PCR result. As part of the limitations of the study, a selection bias was present since the percentage of asymptomatic obstetric patients with a positive RT-PCR result could not be estimated because the universal screening was not performed in our faculty. Furthermore, we were not able to perform an analysis of the rectal or stool samples of the included patients. In the published literature, there are references that support the belief that COVID-19 might be transmitted via the faecal route [29] and may even be a cause of vertical transmission during vaginal delivery in pregnant women with COVID-19, where rectal and stool maternal swabs test positive for SARS-CoV-2 [30]. This diagnostic tool was not available in our hospital during the period of this work, giving rise to a limitation in the interpretation of the results. However, with the current information available, the obstetricians should maintain their obstetrical indications for delivery because contamination does not mean infection or vertical transmission and it is necessary to know the true incidence of this transmission route in large samples of patients [31].

Moreover, an IgG antibody study could not be performed on all the patients. The result of this analysis could have given us more information about which patients had suffered from COVID-19. However, our results should be interpreted with caution and their generalisability may be limited and replicated by more studies.

## 5. Conclusions

In the present study, nearly 39% of the obstetric patients with suspected COVID-19 had a negative RT-PCR. In these patients, the respiratory symptoms and the systemic syndrome were less severe (lower percentage of pneumonia, need for maternal treatment, need for oxygen therapy, admission to the ICU and LDH level, and a higher monocyte count). Only one patient (11%) with pneumonia and a negative RT-PCR result developed SARS-CoV-2 antibodies; therefore, other causes of these cases of pneumonia must be evaluated. Patients with a positive RT-PCR result had a higher proportion of prematurity and C-section. Monocyte count, LDH level and a need for oxygen therapy were the variables related to a negative RT-PCR result. The predictive model created with these variables could help to optimise the resources needed to treat patients with suspected COVID-19 and solve the limitations of RT-PCR, which can be unavailable in some countries around the world.

## Figures and Tables

**Table 1:** Descriptive study of the overall sample and a comparative analysis based on the severe acute respiratory syndrome (SARS-CoV-2) RT-PCR results. Abbreviations: *N*—number of patients, %—percentage, CI—confidence interval, BMI—body mass index, ICU—intensive care unit, NICU: neonatal ICU.

	Overall	RT-PCR Positive	RT-PCR Negative	*p*-Value
*N* (%)	111	68 (61.3%)	43 (38.7%)	
**Maternal Features**				
Maternal race, *n* (%)				0.557
Caucasian	63 (56.8%)	38 (55.9%)	25 (58.1%)	
Hispanic	45 (40.5%)	29 (42.7%)	16 (37.2%)	
Asiatic	3 (2.7%)	1 (1.5%)	2 (4.7%)	
Maternal age, mean (CI 95%)	33.0 (31.8–34.3)	33.5 (32.0–35.0)	32.3 (30.3–34.3)	0.347
Tobacco, *n* (%)	12 (11.1%)	6 (9.1%)	6 (14.3%)	0.408
Maternal morbidities, *n* (%)	45 (40.9%)	25 (36.8%)	20 (47.6%)	0.262
BMI, mean (CI 95%)	26.4 (25.3–27.5)	27.1 (25.6–28.7)	25.3 (23.8–26.9)	0.118
**Obstetric Features**				
Obstetric morbidities, *n* (%)	26 (23.9%)	14 (21.2%)	12 (27.9%)	0.425
Multiparous, *n* (%)	67 (60.9%)	40 (58.8%)	27 (64.3%)	0.568
Onset of symptoms in pregnancy, *n* (%)	93 (84.6%)	57 (83.8%)	36 (85.7%)	0.789
Gestational age at triage, mean (CI 95%)	27.7 (25.5–29.9)	28.6 (25.8–31.4)	26.2 (22.5–29.9)	0.302
**Maternal Signs and Symptoms**				
Fever, *n* (%)	61 (55.0%)	36 (52.9%)	25 (58.1%)	0.591
Cough, *n* (%)	59 (53.2%)	37 (54.4%)	22 (51.2%)	0.738
Shortness of breath, *n* (%)	33 (29.7%)	20 (29.4%)	13 (30.2%)	0.927
Diarrhoea, *n* (%)	7 (6.3%)	4 (5.9%)	3 (7.7%)	0.818
Temperature, mean (CI 95%)	36.7 (36.5–37.0)	36.7 (36.5–37.1)	36.7 (36.5–37.1)	0.884
Oxygen saturation, mean (CI 95%)	96.6 (95.8–97.4)	96.4 (95.7–97.1)	96.9 (95.2–98.5)	0.571
Breathing frequency, mean (CI 95%)	22.1 (19.9–24.2)	23.3 (20.1–26.5)	20.0 (17.9–22.1)	0.139
**Complementary Maternal Studies**				
Pneumonia, *n* (%)	40 (36.4%)	31 (45.6%)	9 (21.4%)	0.009
Leukocytes, mean (CI 95%)	11,465.7 (7963.6–14,967.8)	9762.2 (7255.7–12,268.6)	14,021 (5948.0–22,094.0)	0.239
Lymphocytes, mean (CI 95%)	1351 (1234.6–1467.4)	1301.7 (1160.9–1442.5)	1425 (1218.9–1631.1)	0.306
Monocytes, mean (CI 95%)	690.7 (508.4–872.9)	518.8 (441.0–596.6)	946.2 (508.5–1383.8)	0.022
Platelets, mean (CI 95%)	215,511.8 (195,538.3–235,485.4)	200,453 (178,323.5–222,582.6)	238,100 (200,601.0–275,599.0)	0.067
LDH, mean (CI 95%)	201.6 (182.5–220.6)	224.5 (198.1–250.8)	168 (144.9–191.1)	0.003
**Maternal Treatments**				
Antivirals, *n* (%)	29 (26.4)	24 (35.3%)	5 (11.9%)	0.005
Antibiotics, *n* (%)	36 (32.7%)	29 (42.7%)	7 (16.7%)	0.004
Antirheumatics, *n* (%)	28 (25.5%)	23 (33.8%)	5 (11.9%)	0.008
Anticoagulants, *n* (%)	38 (34.6%)	34 (50.0%)	4 (9.5%)	<0.001
**Maternal–Perinatal Outcomes**				
Oxygen therapy, *n* (%)	20 (18.5%)	19 (28.8%)	1 (2.4%)	<0.001
Admission to ICU, *n* (%)	4 (3.7%)	4 (6.0%)	0 (0.0%)	0.046
C-section	23 (44.2%)	19 (48.7%)	4 (30.8%)	0.253
Prematurity	15 (28.9%)	13 (33.3%)	2 (15.4%%)	0.196
Neonatal birthweight, mean (CI 95%)	2806.1 (2588.2–3025.3)	2755.3 (2487.2–3023.5)	2953.1 (2550.6–3355.5)	0.431
Apgar score at five minutes, mean (CI 95%)	9.5 (9.3-9.8)	9.5 (9.2–9.9)	9.6 (9.2–10.0)	0.865
Admission to NICU, mean (CI 95%)	17 (32.7%)	134 (35.9%)	3 (23.1%)	0.383
Neonatal mortality, *n* (%)	2 (4.2%)	2 (5.7%)	0 (0.0%)	0.255

**Table 2:** COVID-19 severity in the overall sample and in terms of the SARS-CoV-2 RT-PCR results.

COVID-19 Severity	Overall	RT-PCR Positive	RT-PCR Negative	*p*-Value
*N*, (%)	111	68	43	0.022
Mild, *n* (%)	71 (64.0%)	37 (52.4%)	34 (79.1%)	
Moderate, *n* (%)	26 (23.4%)	19 (27.9%)	7 (16.3%)	
Severe, *n* (%)	10 (9.0%)	8 (11.8%)	2 (4.7%)	
Critical, *n* (%)	4 (3.6%)	4 (5.9%)	0 (0.0%)	

## Abbreviations

SARS-CoV-2	severe acute respiratory syndrome coronavirus 2
COVID-19	coronavirus disease 2019
RT-PCR	real-time reverse transcription polymerase chain reaction
ICU	intensive care unit
MPOs	maternal–perinatal outcomes
BMI	body mass index
LDH	lactate dehydrogenase
GA	gestational age
NICU	neonatal intensive care unit
CI	confidence interval
